# The burden, causes, and determinants of blindness and vision impairment in Asia: An analysis of the Global Burden of Disease Study

**DOI:** 10.7189/jogh.14.04100

**Published:** 2024-06-14

**Authors:** Minjie Zou, Aiming Chen, Zhenzhen Liu, Ling Jin, Danying Zheng, Nathan Congdon, Guangming Jin

**Affiliations:** 1 State Key Laboratory of Ophthalmology, Zhongshan Ophthalmic Center, Sun Yat-sen University, Guangdong Provincial Key Laboratory of Ophthalmology and Visual Science, Guangdong Provincial Clinical Research Center for Ocular Diseases, Guangzhou, China; 2 The Fifth Affiliated Hospital of Sun Yat-sen University, Zhuhai, China; 3 Centre for Public Health, Queen’s University Belfast, Belfast, Belfast, UK; 4 Orbis International, New York, New York, USA

## Abstract

**Background:**

Asia accounts for more than half of the world’s population and carries a substantial proportion of the global burden of blindness and vision impairment. Characterising this burden, as well as its causes and determinants, could help with devising targeted interventions for reducing the occurrence of blindness and visual impairment.

**Methods:**

Using the Global Burden of Disease Study 2019 database, we retrieved data on the number of disability-adjusted life years (DALYs); crude and age-standardised rates; and the prevalence (with 95% uncertainty intervals (95%UIs)) of blindness and vision loss due to six causes (age-related macular degeneration, cataracts, glaucoma, near-vision impairment, refractive error, and other vision loss) for Asian countries for the period between 1990 and 2019. We defined DALYs as the sum of the years lost due to disability and years of life lost, and calculated age-standardised figures for the number of DALYs and prevalence by adjusting for population size and age structure. We then evaluated the time trend of the disease burden and conducted subgroup analyses by gender, age, geographic locations, and socio-demographic index (SDI).

**Results:**

In 2019, the DALYs and prevalence of blindness and vision loss had risen by 90.1% and 116% compared with 1990, reaching 15.84 million DALYs (95% UI = 15.83, 15.85) and 506.71 million cases (95% UI = 506.68, 506.74). Meanwhile, the age-standardised rate of DALYs decreased from 1990 to 2019. Cataracts, refractive error, and near vision impairment were the three most common causes. South Asia had the heaviest regional disease burden (age-standardised rate of DALYs = 517 per 100 000 population; 95% UI = 512, 521). Moreover, the burden due to cataracts ranked high in most Asian populations. Being a woman; being older; and having a lower national SDI were factors associated with a greater vision loss burden.

**Conclusions:**

The burden due to vision loss remains high in Asian populations. Cataracts, refractive error, and near vision loss were the primary causes of blindness and vision loss. Greater investment in ocular disease prevention and care by countries with lower socioeconomic status is needed, as well as specific strategies targeting cataract management, women and the elderly.

Vision loss (including blindness and vision impairment) significantly impacts both an individual’s quality of life and a society’s economic development [1–6], making it a significant public health issue. The World Health Organization (WHO) has suggested that at least 2.2 billion people were blind or visually impaired worldwide in 2019, half of whom had conditions that were preventable or treatable [7,8].

Initiatives such as ‘VISION 2020: The Right to Sight’ headed jointly by the International Agency for the Prevention of Blindness and the WHO, along with ‘Towards Universal Eye Health: A Global Action Plan 2014–2019’ led by the World Health Assembly, have provided a framework and impetus for member states to eliminate preventable blindness [9–11]. Yet despite potential modest decreases in the global burden of blindness and vision impairment, existing studies indicate that the specific targets of these plans have not been met in terms of crude prevalence of moderate or worse distance vision [12,13]. Therefore, a basic understanding of the current challenge we are facing is crucial for the development and implementation of future health policies.

With an estimated population of 4.61 billion in 2019, Asia accounts for the majority of the world’s population (7.74 billion in total), meaning it also carries a substantial proportion of the global burden of blindness and vision impairment [14–18]. This burden is expected to increase with the ageing and growing regional population unless further action is taken. In view of these changes, and to assess the impact of global initiatives to restore and protect vision, our knowledge of Asia’s regional burden of blindness and vision loss needs to be updated, while shifts in its specific causes and determinants must be better understood. Identifying similarities and disparities between different sub-regions of Asia may assist in health care policy decision-making and allocation of resources.

In recent decades, studies have found that the burden of blindness and vision loss was consistently high in Asia [14–21]. One study indicated that South Asia had the highest visual impairment prevalence (23.6%; 95% uncertainty interval (UI) = 19.4–29.4%) in 2010, higher than other regions of the world [22]. However, most of these studies either focussed on specific populations in Asia or are currently outdated. A comprehensive and up-to-date evaluation of the region as a whole and with interregional comparisons of blindness and vision loss is needed to inform future strategies.

In this study, we aimed to evaluate the burden of blindness and vision loss using data for the prevalence and disability-adjusted life years (DALYs) in absolute numbers, as well as per-population rates in Asia and its sub-regions between 1990 and 2019. We also investigated various demographic and socioeconomic determinants of vision loss and compared the disease burden among Asian populations with global figures.

## METHODS

### Data sources

The Global Burden of Disease (GBD) Collaborator Group uses all available data on disease occurrence, natural history, and severity, providing it meets predefined inclusion criteria. Within the most recent GBD 2019 study, they evaluated the burden of 369 diseases and injuries in 204 countries and territories since 1990, including blindness and vision loss due to six causes (age-related macular degeneration, cataracts, glaucoma, near vision impairment, refractive error, and other vision loss). The methodology of this study has been described elsewhere [23–25]. In brief, primary data were gathered from various sources, including censuses; household surveys; civil registration and vital statistics; disease registries; health service utilisation records; and others. Using GBD 2019 data (retrieved via the Global Health Data Exchange (GHDx) [26]) for blindness and vision loss, we calculated the disease burden in the terms of DALYs and prevalence for each Asian country and Asia as a whole. To evaluate the disease burden across Asia and assess the variation between different geographical areas, we separated Asia into five geographical sub-regions: Central Asia, East Asia, South Asia, Southeast Asia, and West Asia [27].

We also used the socio-demographic index (SDI), a composite index reflecting a country’s socio-demographic development status, to measure the impact of social development on health care services. This comprehensive indicator ranges from 0 to 1, with higher values indicating a lower total fertility rate of women under 25 years of age and higher per capita income and educational attainment (mean education for those aged 15 and older). Per the SDI, countries are classified into five groups: low (SDI < 0.454), lower-middle (SDI = 0.454–0.606), middle (SDI = 0.607–0.689), upper-middle (SDI = 0.690–0.804), and high SDI (SDI ≥ 0.805) [28].

### Analytic approach

We defined DALYs as the sum of the years lost due to disability (YLD) and years of life lost (YLL) using the following formula:

Number of DALYs = (number of deaths × standard life expectancy at the age of death in years) + (prevalence of condition × disability weight of condition).

We also determined the corresponding uncertainty intervals (UIs), defined as the 2.5% and 97.5% values of the ordered draw [25]. We calculated standard life expectancy from the lowest observed risk of death for each five-year age group in all populations greater than five million [29]. Disability weights were assessed in a previously published study, with the severity of health loss associated with a single given health state [30]. These disability weights are measured on a scale from 0 to 1, with 0 implying a state equivalent to full health and 1 a state equivalent to death. Lastly, we calculated age-standardised figures for the number of DALYs and prevalence by adjusting for population size and age structure.

We retrieved the following data for further analyses: the population of Asian countries in 1990 and 2019; the number of DALYs and prevalence of blindness and vision loss, both overall and cause-specific, among Asian countries in 1990 and 2019; the gender-specific, age-standardised number of DALYs and total and cause-specific prevalence of blindness and vision loss among Asian countries and globally in 1990 and 2019; the age-specific number and age-standardised rate of DALYs due to blindness and vision loss (both total and cause-specific) in Asia in 2019. We used the Wilcoxon sign-rank test to compare differences in DALYs between Asia and the global population.

We performed all analyses in Stata/MP, version 15.1 (StataCorp LLC., College Station, Texas, USA) and generated all figures using the GraphPad Prism softwarem version 5.01 (GraphPad Software, San Diego, California, USA).

## RESULTS

### Overview

The Asian population grew by 40% in 2019 (4.61 billion) compared to 1990 (3.20 billion) and still contributed to a large proportion of the global burden of blindness and vision loss. The prevalence and total DALYs due to blindness and vision loss were 506.71 million (95% UI = 506.68, 506.74) and 15.84 million (95% UI = 15.83, 15.85), respectively (**Table 1**), accounting for 71.0% and 70.2% of the global total. After adjusting for the population and age structure, the age-standardised rate of DALYs and prevalence of blindness and vision impairment per 100 000 population were 376 (95% UI = 372, 380) and 10 930 (95%UI = 10 910–10 950), respectively. Compared to 1990, the total disease burden in terms of DALYs and prevalence of blindness and vision impairment increased up to 2019 by 90.1% and 116%, respectively, though the age-standardised rates decreased by 15.9% and 3.35%.

**Table 1:** Total and regional cause-specific disease burden of blindness and vision impairment for Asia from 1990 to 2019 with 95% UIs in parentheses

	DALYs						Prevalence					
	**Age-standardised rate, 1990**	**Age-standardised rate, 2019**	**Change from 1990 to 2019 (%)**	**Total, 1990**	**Total, 2019**	**Change from 1990 to 2019 (%)**	**Age-standardised rate, 1990**	**Age-standardised rate, 2019**	**Change from 1990 to 2019 (%)**	**Total, 1990**	**Total, 2019**	**Change from 1990 to 2019 (%)**
**Age-related macular degeneration**												
Central Asia	5.17 (4.73, 5.64)	4.34 (4.21, 4.47)	−16.0 (−19.5, −12.9)	1517 (1442, 1595)	2066 (1978, 2157)	36.2 (33.8, 38.7)	76.2 (74.5, 78.0)	75.4 (73.6, 77.1)	−1.14 (−1.41, −0.92)	22 431 (22 139, 22 726)	32 452 (32 100, 32 806)	44.7 (44.0, 45.3)
East Asia	6.72 (6.22, 7.25)	6.48 (5.99, 7.00)	−3.57 (−5.27, −2.30)	59 898 (59 421, 60378)	153 676 (152 914, 154 440)	157 (156, 160)	107 (105, 109)	117 (115, 119)	9.25 (8.71, 9.82)	940 122 (939 213, 942 833)	2 738 260 (2 735 496, 2 741 025)	191 (191, 192)
South Asia	12.5 (11.8, 13.2)	8.69 (8.12, 9.29)	−30.4 (−33.1, −27.9)	62 304 (61 817, 62 794)	112 417 (111 764, 113 072)	80.4 (80.2, 80.7)	152 (149, 154)	119 (117, 121)	−21.8 (−22.5, −21.2)	757 165 (755 216, 758 806)	1 522 937 (1 520 711, 1 525 165)	101 (101, 102)
Southeast Asia	9.27 (8.68, 9.89)	7.53 (7.00, 8.09)	−18.8 (−21.4, −16.3)	19 368 (19 096, 19 642)	38 168 (37 787, 38 552)	97.1 (96.8, 97.3)	97.8 (95.8, 99.7)	83.6 (81.8, 85.4)	−14.5 (−15.2, −13.8)	210 610 (209 721, 211 502)	445 326 (444 048, 446 606)	111 (110, 112)
West Asia	15.6 (14.9, 16.4)	13.7 (13.0, 14.5)	−12.2 (−13.9, −10.6)	16 395 (16 145, 16 648)	35 650 (35 282, 36 021)	117.4 (116.8, 118.2)	174 (171, 176)	182 (179, 185)	4.66 (4.36, 4.99)	186 460 (185 622, 187 300)	448 176 (446 894, 449 460)	140 (139, 141)
Total Asia	9.7 (9.1, 10.3)	7.94 (7.40, 8.51)	−17.9 (−20.5, −15.5)	159 750 (158 974, 160 529)	341 977 (340 851, 343 105)	114.1 (113.9, 114.3)	125 (123, 128)	116 (114, 119)	−7.19 (−7.66, −6.75)	2 116 789 (2 114 257, 2 119 322)	5 187 241 (5 184 144, 5 190 338)	145 (144, 147)
**Cataract**												
Central Asia	81.7 (79.9, 83.5)	83.4 (81.6, 85.2)	2.11 (1.81, 2.44)	23 289 (22 991, 23 590)	29 006 (28 674, 29 341)	24.5 (24.0, 25.1)	1283 (1276, 1290)	1179 (1113, 1248)	−8.11 (−8.26, −7.96)	363 294 (362 135, 364 456)	498 621 (497 273, 499 972)	37.2 (37.0, 37.4)
East Asia	59.0 (57.5, 60.5)	53.5 (52.3, 54.9)	−9.42 (−10.2, −8.69)	492 868 (491 517, 494 211)	1 191 391 (1 189 384, 1 193 400)	142 (141, 143)	787 (781, 792)	899 (893, 905)	14.3 (14.0, 14.5)	6 273 078 (6 270 080, 6 276 075)	19 983 702 (19 975 866, 19 991 543)	218 (217, 219)
South Asia	268.8 (265.6, 272.1)	201 (198, 204)	−25.2 (−25.7, −24.7)	131 6941 (131 4846, 131 9038)	2 571 434 (256 8725, 2 574 144)	93.7 (93.6, 93.9)	3060 (3049, 3071)	2682 (2672, 2692)	−12.4 (−12.5, −12.3)	15 129 638 (15 122 623, 15 136 661)	35 081 936 (35 072 582, 35 091 291)	132 (131, 133)
Southeast Asia	292.8 (289.5, 296.2)	212 (209, 215)	−27.7 (−28.2, −27.1)	618 415 (616 923, 619 910)	1 051 745 (1 049 844, 1 053 648)	70.1 (69.9, 70.2)	3200 (3189, 3211)	2686 (2676, 2696)	−16.1 (−16.2, −15.9)	6 681 814 (6 678 895, 6 684 732)	13 749 190 (13 742 441, 13 755 942)	105 (104, 106)
West Asia	144.4 (142.0, 146.8)	111 (109, 113)	−2.35 (−2.61, −2.11)	126 663 (125 971, 127 358)	236 761 (235 820, 237 705)	86.9 (86.7, 87.1)	1749 (1741, 1758)	1579 (1571, 1586)	−9.76 (−9.90, −9.62)	1 574 836 (1 572 579, 1 577 095)	3 676 594 (3 673 605, 3 679 583)	133 (132, 134)
Total Asia	171.1 (168.5, 173.7)	139 (136, 140)	−19.0 (−19.6, −18.4)	2 578 177 (2 575 466, 2 580 889)	5 080 337 (5 077 238, 5 083 436)	97.1 (97.0, 97.2)	1991 (1982, 1999)	1913 (1904, 1921)	−3.91 (−4.00, −3.83)	30 022 661 (30 013 681, 30 031 657)	72 990 043 (72 973 901, 73 006 213)	143 (142, 144)
**Glaucoma**												
Central Asia	15.8 (15.0, 16.6)	10.8 (10.2, 11.4)	−31.6 (−34.0, −29.3)	4392 (4263, 4524)	4340 (4212, 4471)	−1.18 (−1.55, −0.88)	131 (129, 133)	106 (100, 113)	−18.8 (−19.5, −18.2)	36 669 (36 295, 37 046)	39 691 (39 302, 40 083)	8.24 (7.96, 8.53)
East Asia	9.64 (9.04, 10.3)	5.85 (5.39, 6.34)	−39.3 (−42.5, −36.2)	81 336 (80 780, 81 895)	144 772 (144 033, 145 514)	78.0 (77.7, 78.3)	84.3 (82.5, 86.1)	67.9 (66.3, 69.5)	−19.4 (−20.3, −18.6)	703 982 (702 397, 705 569)	1 643 464 (1 641 168, 1 645 762)	133 (131, 134)
South Asia	17.7 (16.8, 18.5)	11.6 (11.0, 12.3)	−34.1 (−36.4, −31.9)	74 867 (74 334, 75 403)	143 303 (142 567, 144 042)	91.4 (91.2, 91.6)	168 (165, 170)	123 (121, 125)	−26.7 (−27.4, −26.0)	729 728 (728 117, 731 342)	1 540 033 (1 537 796, 1 542 272)	111 (110, 113)
Southeast Asia	13.9 (13.2, 14.7)	10.2 (9.58, 10.8)	−26.8 (−29.2, −24.5)	26 280 (25 964, 26 599)	46 553 (46 132, 46 977)	77.1 (76.6, 77.8)	118 (116, 120)	92.5 (90.6, 94.4)	−21.6 (−22.4, −20.9)	225 749 (224 829, 226 672)	441 118 (439 846, 442 393)	95.4 (95.3, 95.5)
West Asia	34.4 (33.3, 35.6)	27.0 (26.0, 28.1)	−21.6 (−23.0, −20.2)	32 922 (32 568, 33 279)	54 891 (54 434, 55 351)	66.7 (66.2, 67.2)	270 (267, 273)	209 (206, 212)	−22.6 (−23.1, −22.1)	255 588 (254 611, 256 568)	474 132 (472 816, 475 451)	85.5 (85.3, 85.7)
Total Asia	14.8 (14.1, 15.6)	10.4 (9.82, 11.1)	−29.5 (−31.0, −27.2)	219 796 (218 888, 220 707)	373 585 (372 410, 374 762)	70.0 (69.7, 70.2)	131 (129, 134)	105 (103, 107)	−20.3 (−21.0, −19.6)	1 951 716 (1 949 260, 1 954 174)	4 138 438 (4 135 385, 4 141 491)	112 (111, 113)
**Near-vision impairment**												
Central Asia	68.6 (67.0, 70.2)	65.7 (64.2, 67.4)	−4.16 (−4.65, −3.70)	21 248 (20 964, 21 535)	33 099 (32 744, 33 457)	55.8 (55.1, 56.4)	6905 (6889, 6921)	6607 (6131, 7115)	−4.31 (−4.36, −4.26)	2 132 486 (2 129 948, 21 35 026)	3 306 163 (3 303 247, 3 309 080)	55.0 (54.8, 55.1)
East Asia	66.4 (64.8, 68.1)	66.2 (64.6, 67.8)	−0.36 (−0.54, −0.23)	675 826 (674 271, 677 384)	1 598 693 (1 596 422, 1 600 966)	137. (136, 138)	6684 (6669, 6700)	6627 (6612, 6643)	−0.85 (−0.88, −0.83)	67 438 056 (67 428 877, 674 472 444)	15 956 1618 (15 953 8922, 15 958 4334)	137 (136, 137)
South Asia	111.7 (109.6, 113.8)	106 (104, 108)	−4.82 (−5.23, −4.43)	705 341 (703 755, 706 930)	1 623 009 (1 620 724, 1 625 296)	130 (129, 131)	11 417 (11 397, 11 436)	10 810 (10 790, 10 821)	−5.32 (−5.36, −5.28)	71 303 026 (71 294 160, 71 311 891)	164 121 775 (164 098 800, 164 144 718)	130 (130, 131)
Southeast Asia	50.6 (49.3, 50.2)	45.6 (44.3, 46.9)	−10.0 (−10.9, −9.20)	121 267 (120 590, 121 947)	266 365 (265 368, 267 365)	120 (119, 121)	5165 (5151, 5178)	4635 (4622, 4648)	−10.3 (−10.3, −10.2)	12 229 876 (12 223 466, 122 363 004)	26 832 054 (26 823 371, 26 840 744)	119 (118, 120)
West Asia	38.4 (37.2, 39.7)	32.2 (31.1, 33.3)	−16.2 (−17.4, −15.1)	43 501 (43 094, 43 911)	92 716 (92 123, 93 312)	113 (112, 114)	3917 (3905, 3929)	3242 (3231, 3253)	−17.2 (−17.4, −17.1)	4 380 843 (4 377 768, 4 383 919)	9 366 727 (9 387 240, 9 390 211)	114 (113, 115)
Total Asia	78.2 (76.5, 80.0)	76.0 (74.3, 77.7)	−2.89 (−3.28, −2.53)	1 567 182 (1 564 929, 1 569 437)	3 613 882 (3 610 904, 3 616 860)	131 (130, 132)	7947 (7940, 7964)	7682 (7665, 7698)	−3.34 (−3.38, −3.30)	157 484 287 (1 574 617 399, 157 506 912)	363 188 337 (363 158 501, 363 218 123)	131 (130, 132)
**Refractive error**												
Central Asia	70.2 (68.5, 71.8)	64.2 (62.7, 65.8)	8.47 (−9.14, −7.82)	28 564 (28 234, 28 897)	42 436 (42 034, 42 841)	48.6 (48.0, 49.1)	1819 (1811, 1828)	1727 (1647, 1810)	−5.10 (−5.20, −4.99)	755 232 (752 595, 756 871)	1 128 387 (1 126 427, 1 130 350)	49.4 (49.3, 49.5)
East Asia	69.6 (68.0, 71.2)	66.8 (65.2, 68.4)	−4.04 (−4.53, −3.59)	78 6136 (78 4469, 787 806)	1 407 540 (1 406 384, 1 410 698)	79.1 (78.9, 79.2)	1504 (1496, 1511)	1498 (1491, 1506)	−0.37 (−0.40, −0.34)	17 339 017 (17 331 601, 19 346 428)	30 663 049 (30 654 011, 30 672 092)	76.8 (76.6, 76.9)
South Asia	200.8 (198.0, 203.6)	144 (142, 147)	−28.0 (−28.6, −27.4)	1 419 916 (1 417 753, 1 422 081)	2 276 281 (2 273 682, 2 278 881)	60.3 (60.2, 60.4)	4475 (4462, 4488)	3437 (3425, 3448)	−23.2 (−23.3, −23.1)	33 048 641 (33 039 424, 33 057 869)	54 758 047 (54 748 299, 54 767 800)	65.7 (65.4, 65.9)
Southeast Asia	79.7 (78.0, 81.5)	70.0 (68.4, 71.7)	−12.1 (−12.9, −11.4)	288 301 (287 265, 289 340)	438 784 (437 515, 440 055)	52.2 (52.0, 52.4)	1959 (1951, 1968)	1793 (1785, 1802)	−8.47 (−8.60, −8.35)	7 425 330 (7 422 619, 7 428 040)	11 286 319 (11 280 121, 11 292 523)	52.0 (51.5, 52.6)
West Asia	103 (101, 105)	102 (99.8, 1034)	−0.82 (−1.01, −0.65)	186 562 (185 724, 187 403)	338 101 (336 982, 339 223)	81.2 (81.0, 81.4)	2351 (2342, 2361)	2274 (2265, 2284)	−3.27 (−3.35, −3.20)	4 406 068 (4 402 991, 4 409 146)	8 138 353 (8 135 939, 8 140 765)	84.7 (84.4, 84.9)
Total Asia	119 (117, 121)	100 (98.1, 102)	−16.0 (−16.7, −15.4)	2 709 478 (2 706 723, 2 712 234)	4 503 141 (4 500 057, 4 506 225)	66.2 (66.1, 66.3)	2670 (2660, 2680)	2364 (2354, 2374)	−11.5 (−11.6, −11.3)	62 974 287 (62 964 822, 62 983 754)	105 974 154 (105 955 122, 1 059 932 045)	68.2 (68.1, 69.4)
**Other vision loss**												
Central Asia	75.2 (73.5, 76.9)	58.68 (57.19, 60.20)	−21.9 (−22.9, −21.0)	24 150 (23 847, 24 456)	30 743 (30 401, 31 088)	27.3 (26.7, 27.9)	928 (923, 934)	825 (797, 883)	−11.2 (−11.4, −10.9)	294 145 (293 099, 295 194)	406 560 (405 337, 407 786)	38.2 (38.0, 38.4)
East Asia	33.2 (32.1, 34.4)	28.96 (27.91, 30.03)	−12.9 (−14.0, −11.7)	362 271 (361 114, 363 431)	646 824 (64 530, 64 835)	78.5 (78.4, 78.7)	341 (337, 344)	336 (332, 339)	−1.40 (−1.53, −1.28)	3 573 558 (3 570 588, 3 576 529)	7 642 590 (7 642 659, 7 647 919)	113 (112, 115)
South Asia	54.8 (53.3, 56.2)	44.63 (43.33, 45.96)	−18.5 (−19.5, −17.5)	357 368 (356 218, 358 520)	649 852 (648 325, 651 382)	81.8 (81.7, 82.0)	691 (686, 696)	599 (594, 603)	−13.3 (−13.6, −13.1)	4 33 2291 (4 329 219, 4 335 363)	8 776 210 (8 774 177, 8 778 241)	102 (101, 104)
Southeast Asia	95.2 (93.3, 97.1)	72.38 (70.72, 74.07)	−24.0 (−24.8, −23.1)	253 209 (252 236, 254 185)	413 399 (412 166, 414 635)	63.3 (63.1, 63.5)	1015 (1009, 1021)	807 (802, 813)	−20.5 (−20.7, −20.2)	2 604 854 (2 602 134, 2 607 575)	4 696 782 (4 693 688, 4 699 876)	80.3 (80.1, 80.5)
West Asia	65.4 (63.9, 67.0)	51.08 (49.69, 52.50)	−21.9 (−23.0, −21.0)	99 202 (98 589, 99 818)	163 050 (162 266, 163 837)	64.4 (64.1, 64.7)	599 (554, 564)	553 (549, 558)	−7.69 (−7.91, −7.48)	930 431 (928 631, 932 233)	1 683 360 (1 681 041, 1 685 681)	80.9 (80.8, 81.0)
Total Asia	52.2 (50.8, 53.6)	43.07 (41.79, 44.38)	−17.5 (−18.6, −16.5)	1 096 200 (1 094 264, 1098138)	1 903 868 (1 901 435, 1 906 303)	73.7 (73.6, 73.8)	584 (579, 588)	530 (525, 534)	−9.25 (−9.49, −9.02)	11 735 279 (11 728 971, 11 741 599)	23 205 502 (23 197 231, 23 213 788)	97.7 (97.7, 97.8)
**Total blindness and vision impairment**												
Central Asia	317 (313, 320)	288.53 (285.21,291.86)	−8.85 (−9.17, −8.54)	103 159 (102 534, 103 787)	141 690 (140 958, 142 424)	37.3 (37.0, 37.6)	9756 (9737, 9774)	9302 (9123, 9483)	−4.65 (−4.69, −4.61)	3 217 578 (3 214 683, 3 220 474)	4 946 506 (4 943 407, 4 949 605)	53.7 (53.4, 53.9)
East Asia	245 (242, 248)	227.72 (224.78, 230.69)	−6.92 (−7.24, −6.60)	2 458 334 (2 455 666, 2 461 004)	5 142 896 (5 139 798, 5 145 994)	109 (108, 109)	8575 (8558, 8593)	8676 (8659, 8694)	1.18 (1.15, 1.20)	88 913 654 (88 907 501, 88 919 812)	203 211 571 (203 186 613, 203 236 598)	129 (128, 130)
South Asia	666 (661, 671)	516.90 (512.46, 521.36)	−22.4 (−22.7, −22.1)	3 936 738 (3 933 710, 3 939 76)	7 379 297 (7 376 570, 7 382 022)	87.5 (87.4, 87.6)	16 048 (16 025, 16 071)	14 833 (14 811, 14 855)	−7.58 (−7.62, −7.53)	105 842 685 (105 823 613, 105 86 1801)	226 385 150 (226 359 202, 226 411 121)	113 (111, 115)
Southeast Asia	542 (537, 546)	417.55 (413.56, 421.57)	−22.9 (−23.3, −22.6)	1 327 109 (1 325 007, 1 329 213)	2 255 013 (2 252 423, 2 257 604)	69.9 (69.8, 70.0)	9575 (9557, 9594)	8596 (8579, 8613)	−10.23 (−10.28, −10.18)	25 683 929 (25 675 372, 25 692 494)	50 440 868 (50 431 071, 50 450 678)	96.4 (96.2, 96.5)
West Asia	401 (397, 405)	336.81 (333.23, 340.42)	−16.0 (−16.4, −15.6)	505 246 (504 069, 506 786)	9 21 1 68 (919 376, 922 962)	82.3 (82.2, 82.4)	7875 (7858, 7892)	7103 (7087, 7119)	−9.80 (−9.87, −9.73)	10 688 158 (10 682 101, 10 694 224)	21 722 042 (21 713 964, 21 730 139)	103 (101, 107)
Total Asia	445 (441, 449)	376.09 (372.31, 379.90)	−15.5 (−15.9, −15.2)	8 330 585 (8 328 272, 8 332 896)	1 583 7064 (1 582 9910, 1 584 5220)	90.1 (90.0, 90.1)	11 302 (11 282, 11 322)	10 927 (10 908, 10 947)	−3.32 (−3.35, −3.28)	234 346 004 (234 319 823, 234 372 398)	506 706 138 (506 675 216, 506 737 179)	116 (114, 117)

DALY – disability-adjusted life years

### Regional discrepancy

South Asia had the highest burden among all Asian sub-regions. It had the highest age-standardised rate of DALYs per 100 000 population (517; 95% UI = 512, 521), while East Asia had the lowest (228; 95% UI = 225, 231) (**Table 1**). For the age-standardized figures, South Asia ranked first (14830, 95%UI: 14810, 14850 per 100 000 population), while West Asia ranked last (7100, 95%UI: 7087, 7119 per 100 000 population).

### Primary causes of disease burden

**Figure 1:**
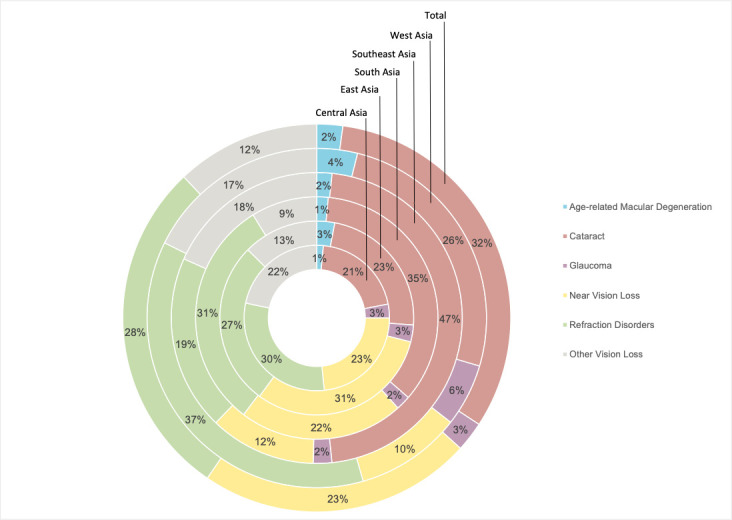
Contribution of age-related macular degeneration, cataracts, glaucoma, near vision loss, refraction disorders and other vision loss to the overall DALYs in central, east, south, southeast, west and total Asia in 2019.

Cataracts (5 080 000; 95% UI = 5 070 000, 5 083 000) were the most common cause of blindness and vision impairment in Asia, followed by refractive error (4 503 000; 95% UI = 4 500 000, 4 506 000) and near vision impairment (3 614 000; 95% UI = 3 611 000, 3 617 000) in terms of the burden of DALYs, accounting for 32%, 28% and 23% of total DALYs, respectively (**Figure 1****, ****Table 1**). However, near vision loss surpassed cataracts as the leading cause of blindness and vision loss in terms of absolute prevalence, as it affected more people, but had a lower impact on disability. West Asia had the greatest regional burden of age-related macular degeneration and glaucoma when comparing age-standardised rates. In contrast, Southeast Asia had the highest burden in terms of cataracts, while South Asia ranked first in near vision loss and refractive disorders (**Table 1**).

### Gender discrepancy

We found substantial gender differences in the burden of blindness and vision loss in Asia. The age-standardised rate of DALYs among women was greater in Asia as a whole (men: 349 per 100 000 population; 95% UI = 345, 352 vs women: 392 per 100 000 population; 95% UI = 389, 397) and in all regions except West Asia (Table S1 in the **Online Supplementary Document**). We also observed this greater burden among women for prevalence in Asia as a whole (men: 10 350 per 100 000 population; 95% UI = 103 330, 10 370 versus women: 11 460 per 100 000 population; 95% UI = 11 440, 11 480).

### Socioeconomic difference

We found that the degree of development may impact the burden of blindness and vision loss. Generally speaking, countries with higher SDI had a lower disease burden of blindness, as well as vision loss of all causes between 1990 and 2019 when compared to those with lower SDI (**Table 2**).

**Table 2:** Age-standardised rate of DALYs for blindness and vision loss by SDI from 1990 to 2019 with 95% UIs in parentheses

	Low SDI countries			Lower-middle SDI countries			Middle SDI countries			Upper-middle SDI countries			High SDI countries		
	**1990**	**2019**	**Change (%)**	**1990**	**2019**	**Change (%)**	**1990**	**2019**	**Change (%)**	**1990**	**2019**	**Change (%)**	**1990**	**2019**	**Change (%)**
**Age-related macular degeneration**	14.8 (14.1, 15.6)	13.8 (13.1, 14.6)	−6.49 (−7.87, −5.29)	11.7 (11.1, 12.4)	7.87 (7.32, 8.42)	−32.8 (−35.6, −30.2)	8.39 (7.83, 8.98)	7.48 (6.96, 8.03)	−10.9 (−13.2, −8.8)	13.3 (12.6, 14.0)	12.3 (11.6, 13.0)	−7.44 (−8.98, −6.09)	2.74 (2.43, 3.08)	2.82 (2.50, 3.17)	2.92 (2.71, 5.67)
Cataract	268 (265, 271)	237 (235, 240)	−11.5 (−11.9, −11.1)	263 (257, 266)	190 (187, 192)	−27.7 (−28.3. −27.2)	123 (121, 125)	102 (100, 104)	−17.0 (−17.7, −16.3)	145 (142, 147)	117 (115, 119)	−19.1 (−19.77, −18.5)	22.8 (21.9, 23.8)	24.2 (23.3, 25.2)	6.18 (5.23, 7.25)
Glaucoma	14.9 (14.2, 15.7)	13.8 (13.1, 14.6)	−7.36 (−8.81, −6.09)	18.1 (17.3, 19.0)	11.7 (11.1, 12.4)	−35.4 (−37.6, −33.2)	12.9 (12.2, 13.6)	8.91 (8.34, 9.51)	−31.2 (−33.8, −28.6)	21.5 (20.6, 22.4)	15.6 (14.8, 16.4)	−27.3 (−29.2, −25.4)	7.81 (7.27, 8.38)	6.61 (6.12, 7.13)	15.4 (12.9, 18.1)
Near,vision impairment	90.9 (89.0, 92.8)	77.2 (75.6, 78.9)	−15.0 (−15.8, −14.3)	110 (108, 112)	106 (104, 108)	−3.96 (−4.34, −3.61)	67.0 (65.4, 68.6)	63.2 (61.7, 64.7)	−5.62 (−6.19, −5.08)	45.0 (43.7, 46.3)	41.3 (40.0, 42.5)	−8.23 (−9.07, −7.44)	12.2 (11.5, 12.9)	13.0 (12.3, 13.8)	7.07 (5.69, 8.65)
Refractive error	125 (123, 128)	128 (126, 131)	2.39 (2.12, 2.66)	202 (199, 205)	143 (141, 145)	−29.0 (−29.6, −28.3)	76.2 (74.5, 78.0)	70.9 (69.4, 72.6)	−6.94 (−7.53, −6.38)	88.0 (86.2, 89.9)	82.4 (80.7, 84.1)	−6.37 (−6.90, −5.87)	48.3 (46.9, 49.6)	48.6 (47.4, 49.9)	0.66 (0.45, 0.93)
Other vision loss	50.6 (49.2, 52.0)	45.9 (44.7, 47.2)	−9.22 (−10.1, −8.44)	54.9 (53.4, 56.3)	44.6 (43.4, 45.8)	−18.6 (−19.7, −17.6)	53.4 (52.0, 54.8)	44.4 (43.5, 45.8)	−16.8 (−17.8, −15.8)	62.9 (61.3, 64.5)	49.4 (48.1, 50.8)	−21.4 (−22.4, −20.4)	21.5 (20.6, 22.4)	19.2 (18.4, 20.1)	−10.5 (−11.9, −9.24)
Total blindness and vision impairment	565 (560, 569)	517 (513, 520)	−8.53 (−8.76, −8.30)	659 (654, 665)	503 (500, 506)	−23.7 (−24.0, −23.3)	341 (337, 344)	297 (294, 299)	−12.9 (−13.2, −12.5)	375 (371, 379)	318 (315, 321)	−15.1 (−15.5, −14.8)	115 (113, 117)	114 (112, 116)	−6.94 (−8.63, −5.50)

DALY – disability-adjusted life year, SDI – socio-demographic index

### Change of disease burden with age

**Figure 2:**
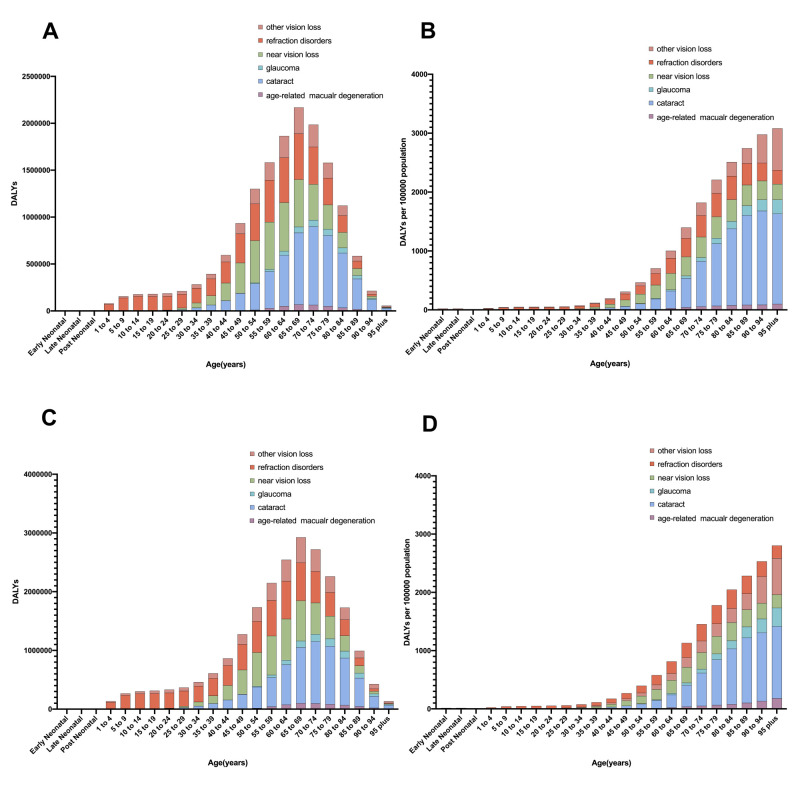
Age-specific DALYs number and rate of blindness and vision loss in 2019 among Asian and global population. **Panel A.** DALYs number among Asian population. **Panel B.** Rate of DALYs among Asian population. **Panel C.** DALYs number among global population. **Panel D.** Rate of DALYs among global population.

Regarding the age-stratified disease burden of blindness and vision loss in 2019, the maximum DALY burden occurred in individuals aged 65 to 69 years both in Asia **(****Figure 2**, Panels A and B**)** and worldwide **(****Figure 2**, Panels C and D). Meanwhile, the age-specific rate of DALYs increased with age, both globally and in Asia. The leading cause of vision loss gradually shifted from refractive error to cataracts after the age of 40 years. The age-standardised burden of DALYs was greater in Asia than globally for cataracts (*P* = 0.001), near vision loss (*P* < 0.001), and refractive error (*P* < 0.001), but lower for glaucoma (*P* = 0.004).

## DISCUSSION

In this study, we gave a comprehensive view of the burden of blindness and vision loss in terms of prevalence and DALYs in Asia between 1990 and 2019. The total burden of vision loss has increased, as Asia’s population has grown and aged. However, due to coordinated national efforts, the risk of vision loss for any particular person (represented by age-adjusted rates) has decreased over the last 30 years. We found that, among the five Asian regions, South Asia had the highest burden in terms of DALYs and prevalence after adjusting for population and age structure. This is consistent with a previous study using the GBD database, which also showed that South Asia accounted for one-third of global blindness in 2015 [17]; this study also indicated that a total of 11.76 million people (32.65% of the worldwide total) experienced blindness due to various ocular diseases.

Though we observed interregional discrepancies, the age-adjusted rate of disease burden due to blindness and vision loss in Asia has had a decreasing trend in the past decades. In general, the overall economic improvement among developed and developing countries and regions in Asia, alongside efforts aimed at ensuring increased accessibility to eye care, as well as a sufficient number of facilities and eye care providers, and better health care coverage [30], have led to improved ocular disease outcomes.

The age-standardised rate of disease burden were decreasing while we are still facing severe challenges due to the large population of Asia. While the patterns of cause-specific vision loss varied substantially by sub-regions, we observed thrends that were consistent across Asia as a whole. Women, older persons, and those residing in countries with a lower SDI had a heavier disease burdens.

Specifically, we found that women suffered a heavier burden of blindness and vision loss compared to men, which is consistent with previous reports [30–32]. Women have a greater life expectancy, leading to a higher risk of developing age-related ocular diseases such as cataracts, near vision loss and age-related macular degeneration [33–35]. Anatomical (as with angle-closure glaucoma) and hormonal (as with cataracts) factors may also play a role [36,37]. Moreover, prior work has concluded that inequitable access to eye care services is also an important determinant of the additional burden of blindness and vision impairment among women [12]. Our results highlight the need for programmes to improve women’s access to eye care services throughout Asia. For example, community outreach vision screening services could help improve equity, particularly for conditions like cataracts and glaucoma, by ensuring improved access to eye care services for women [12].

The leading causes of blindness and vision loss all increase with age. Here we presume that population ageing is a key reason for why the total disease burden for Asia rose, while the age-standardised rate of DALYs and prevalence declined between 1990 and 2019. Recently, the world’s population of people aged 65 years or above surpassed the number of those under the age of 5 years for the first time [13,38]. Vision loss places a particular burden on the elderly, including an increased risk of falls [39,40] and increased all-cause mortality [12,41]. Moreover, improved diagnostic surveillance and updated diagnostic tools may explain the change in the disease burden of blindness and vision loss [42,43]. Governments in Asia need to invest further in initiatives to increase access to and uptake of eye care services among older persons. Concerning this, health technology assessments are intended to be implemented for evaluating the cost-effectiveness of treating different ocular diseases in the elderly. This is crucial due to constrained resources and the significant challenge of addressing potentially unlimited demands, leading to the necessity of making choices and often rationing services [44].

We also saw that countries with a lower SDI had higher burdens of blindness and vision loss. This presumably reflects a shortage of material and human resources to deliver vision care in low SDI countries, a determinant of blindness which is well-described [45–48]. As noted above, effective and low-cost treatments for the most important causes of vision loss in Asia, including refractive error, cataracts, and near vision loss, could be helpful in this regard. Consequently, we hope that investment in these low-cost treatments across the region will be spurred by increasing evidence of a very high return on investment and significant gains in economic productivity [49–51]. Moreover, increased economic and education levels were found to enhance awareness and increase the utilisation of eye care services [12]. Lastly, improving the availability of cataract and refractive services in resource-limited settings may contribute to the earlier identification and management of early glaucoma and retinal diseases that would have an irreversible impact on visual health.

The most readily treatable causes of blindness and vision loss – cataracts and refraction disorders [10,52] – accounted for the majority of the disease burden among Asian populations, which is consistent with previous studies [14–19,21]. Relatedly, proven, safe, and low-cost methods exist to treat these conditions, namely cataract surgery and spectacles [53,54]. Despite this, the rate of cataract surgeries remains relatively low in many parts of Asia [55–57], with epidemics of myopia present in many subregions, particularly East Asia [58]. Ambitious initiatives such as India’s ‘National Programme for Control of Blindness and Visual Impairment’ [59], China’s ‘Sight First China Action’ [18] and others [60,61] have focused on this problem; in fact, rates for cataract surgeries in some Asian countries such as India have been approaching those of high-income countries. The reason for the high blindness and vision loss burden due to cataracts may lie in the fact that cataract surgery must be performed by a trained surgeon in a facility with the capacity to conduct operations and handle postoperative complications. Consequently, the imbalance of regional development leads to a geographically unequal distribution of surgeries performed by adequate surgeons with proper equipment [62,63]. Sending experienced surgeons and surgical equipment to remote areas and providing training programmes for junior ophthalmologists in rural regions to enhance surgical service capacity has now become a trend in developing countries [64–66]. Regarding refractive errors, China launched a national myopia management programme in 2018, coordinating activities of multiple ministries with the aggressive target of annual reductions of 0.5% in myopia prevalence [67].

Being historically underaddressed, near vision loss is another important cause of blindness and vision loss. The definition used by the GBD [68,69] indicates that this is a reversible condition, readily correctable at low cost and high efficacy with glasses. The fact that the burden of vision impairment and associated disability remains so high, even in Asia’s rapidly developing countries, signifies that more must be done to target this problem. The importance of the problem is underscored by high-quality evidence showing the impact of near-vision impairment on workplace productivity [50]. Studies have also suggested that lower educational levels and a lack of private health insurance are associated with higher risk of near vision loss [70,71].

One strength of this study is the inclusion of data from across the sub-regions of Asia using a standardised and widely-accepted approach [25]. We must also acknowledges ome limitations, most of which are similar to those in the GBD study – most notably the restricted data availability and statistical assumptions [24]. Besides this, the disease burden of blindness and vision loss could be underestimated in districts with inadequate ophthalmologic resources to carry out epidemiological research. Possible solutions may be to actively engage with local communities, health care providers, and authorities, and synergistically gather more comprehensive and precise data on the prevalence and impact of ocular diseases. The current digital technologies, telemedicine, and artificial intelligence may have promising roles in evaluating the disease burden in these remote districts. Additionally, future studies could help us better understand the cost-effectiveness evaluation of different strategies and policies for screening and treating ocular diseases.

Nevertheless, our analysis is the first to report the overall disease burden in terms of DALYs and the prevalence of blindness and vision loss among the Asian population. Our findings underscore the urgency for targeted policies and strategies to address the specific challenges faced by Asian regions, especially South Asia, in combating visual impairment. Here we offer specific advice for informing policies and setting priorities for action, including the need for initiatives targeting women and the elderly, but also to invest in low-cost care with high rates of return and proven impact on improved outcomes, such as cataract surgery and near and distance refractive services.

## CONCLUSIONS

The burden of blindness and vision loss remains high in Asian populations. Preventable blindness, such as cataracts and refractive errors, which contribute to a large proportion of the burden, could be solved at a relatively low cost. We urge policymakers to carefully consider these issues. As subgroups of lower socioeconomic status, women and the elderly bear a significantly heavier disease burden, necessitating focussed prevention for these populations.

## Additional material


Online Supplementary Document
