# Supplementary material for: The burden, causes, and determinants of blindness and vision impairment in Asia: An analysis of the Global Burden of Disease Study
Source: J Glob Health. 2024 Jun 14;14:04100. doi: 10.7189/jogh.14.04100 (PMC11170234; doi:10.7189/jogh.14.04100)
Supplement: Online Supplementary Document [file jogh-14-04100-s001.pdf]

Table S1. Burden of blindness and vision loss stratified by gender in the global and total and sub-regional Asian populations in 2019.

	DALYs			Prevalence		
	**Men**	**Women**	**Total**	**Men**	**Women**	**Total**
**Age-Related Macular Degeneration**						
**Central Asia**	6.47 (6.31, 6.63)	4.30 (4.17, 4.43)	4.34 (4.21, 4.47)	48.6 (47.2, 50.0)	48.6 (47.3, 50.0)	75.3 (73.6, 77.1)
**East Asia**	5.74 (5.28, 6.23)	7.09 (6.58, 7.63)	6.48 (5.99, 7.00)	107 (105, 109)	125 (123, 127)	117 (114.9, 119.)
**South Asia**	8.41 (7.85, 9.00)	8.97 (8.39, 9.58)	8.69 (8.12, 9.29)	155 (153, 158)	122 (120, 124)	119 (117, 121)
**Southeast Asia**	6.80 (6.30, 7.33)	8.08 (7.53, 8.66)	7.53 (7.00, 8.09)	81.2 (79.5, 83.0)	85.5 (83.7, 87.3)	83.6 (81.8, 85.4)
**West Asia**	8.80 (8.23, 9.40)	13.7 (12.9, 14.4)	13.7 (13.0, 14.5)	169 (166, 173)	195 (192, 198)	182 (179,185)
**Asia**	7.07 (6.56, 7.61)	8.32 (7.76,8.91)	7.94 (7.40, 8.51)	110 (107, 113)	122 (119, 124)	116 (114, 119)
**Global**	6.24 (4.33, 8.70)	7.66 (5.35, 10.7)	7.05 (4.92, 9.84)	89.5 (74.5, 105)	102 (86.1, 112)	96.8 (81.3, 113)
**Cataract**						
**Central Asia**	61.4 (59.9, 62.9)	84.1 (82.3, 85.9)	83.4 (81.6, 85.2)	1078 (1015,1144)	1074 (1011,1140)	1179 (1113, 1248)
**East Asia**	44.9 (43.5, 46.2)	59.8 (58.3, 61.3)	53.5 (52.0, 54.9)	760 (755, 766)	1007 (1001, 1013)	899 (893, 905)
**South Asia**	179 (177, 182)	222 (219, 225)	201 (198, 204)	2447 (2437, 2456)	2914 (2904, 2925)	2682 (2672, 2692)
**Southeast Asia**	178 (175, 180)	238 (235, 241)	212 (209, 215)	2371 (2362, 2380)	2928 (2918, 2938)	2686 (2676, 2696)
**West Asia**	87.1 (85.3, 88.9)	88.5 (86.6, 90.3)	111 (109, 113)	1423 (1415, 1430)	1734 (1726, 1742)	1579 (1571, 1586)
**Total**	120 (117, 122)	151 (149, 153)	139 (136, 141)	1711 (1703, 1719)	2089 (2081, 2098)	1913 (1904, 1921)
**Global**	74.0 (53.3, 101)	89.8 (63.9, 121)	82.9 (59.1, 112)	1097 (965, 1237)	1302 (1150, 1470)	1208 (1065, 1361)
**Glaucoma**						

**Central Asia**	12.6 (11.9, 13.3)	12.6 (11.9, 13.3)	10.8 (10.2, 11.5)	123 (116, 130)	123 (116, 130)	106 (100, 113)
**East Asia**	6.70 (6.20, 7.23)	5.19 (4.75, 5.66)	5.85 (5.39, 6.34)	73.9 (72.2, 75.6)	63.4 (61.8, 64.9)	67.9 (66.3, 69.5)
	13.9 (13.1, 14.6)	9.67 (9.07, 10.3)	11.6 (11.0, 12.3)	144 (141, 146)	105 (103, 107)	123 (121, 125)
**Southeast Asia**	10.9 (10.2, 11.6)	9.71 (9.11, 10.3)	10.2 (9.58, 10.9)	98.8 (96.9, 101)	87.9 (86.1,89.9)	92.5 (90.6, 94.4)
**West Asia**	19.5 (18.6, 20.4)	15.5 (14.8, 16.3)	27.0 (26.0, 28.0)	236 (233, 239)	182 (179.6, 185)	209 (206, 212)
**Total**	11.1 (10.4, 11.7)	8.44 (7.88, 9.03)	10.4 (9.82, 11.1)	118 (116, 121)	93.2 (91.4, 95.2)	105 (102, 107)
**Global**	11.3 (7.81, 15.7)	8.18 (5.64, 11.4)	9.52 (6.58, 13.2)	108 (91.9, 126)	84.4 (71.3, 99.4)	94.7 (80.5, 111)
**Near Vision Impairment**						
**Central Asia**	65.8 (64.2,67.4)	64.6 (63.0, 66.2)	65.7 (64.2, 67.4)	6214 (6199, 6229)	6211 (6062, 6362)	6607 (6131, 7115)
**East Asia**	62.0 (60.5, 63.6)	70.1 (68.4, 71.7)	66.2 (64.6, 67.8)	6166 (6151, 6181)	7046 (7031, 7062)	6627 (6612, 6643)
**South Asia**	101.4 (99.4, 103.4)	111 (109, 113)	106 (104.3, 108)	10248 (10229, 10267)	11364 (11345, 11384)	10810 (10790, 10821)
**Southeast Asia**	42.4 (41.1, 43.6)	48.2 (46.9, 49.6)	45.6 (44.3,46.9)	4294 (4281, 4307)	4915 (4902, 4928)	4635 (4622, 4648)
**West Asia**	50.1 (48.7, 51.5)	22.1 (21.2, 23.1)	32.2 (31.1, 33.3)	3082 (3071, 3092)	3402 (3391, 3413)	324 (3231, 3253)
**Total**	73.7 (72.0, 75.4)	78.8 (77.1, 80.6)	76.0 (74.3, 77.7)	7228 (7212, 7244)	8096 (8079, 8113)	7682 (7665, 7698)
**Global**	56.0 (25.7, 111)	61.7 (28.3, 122)	58.9 (27.1, 116)	5612 (4091, 7377)	6236 (4558, 8128)	5938 (5336, 7772)
**Refractive Error**						
**Central Asia**	65.5 (63.9, 67.1)	58.4 (56.9, 59.9)	64.2 (62.7, 65.8)	1612 (1535, 1692)	1612 (1587, 1637)	1727 (1647, 1810)
**East Asia**	61.8 (60.3, 63.4)	71.5 (69.8, 73.2)	66.8 (65.2, 68.4)	1383 (1376,1390)	1610 (1602, 1617)	1498 (1491, 1506)
**South Asia**	138 (136, 141)	151 (148, 153)	144 (142, 147)	3307 (3296, 3319)	3576 (3565, 3588)	3437 (3425, 3447)
**Southeast Asia**	70.8 (69.2, 72.5)	69.7 (68.1, 71.4)	70.0 (68.4, 71.7)	1794 (1786, 1802)	1804 (1796, 1812)	1793 (1785, 1802)
**West Asia**	83.1 (81.3, 84.9)	96.5 (94.6, 98.5)	102 (99.8, 104)	2225 (2216, 2234)	2327 (2318, 2337)	2274 (2265, 2284)
**Total**	94.5 (92.6, 96.4)	103 (101, 106)	100 (98.1, 102)	2268 (2258, 2277)	2461 (2451, 2470)	2364 (2355, 2374)

**Global**	77.9 (52.4, 110)	85.0 (57.5, 119)	81.5 (54.9, 115)	1873 (1673, 2082)	2046 (1829, 2275)	1960 (1751, 2179)
**Other Vision Loss**						
**Central Asia**	56.0 (54.6, 57.5)	56.2 (54.8, 57.7)	58.7 (57.2, 60.2)	805 (750, 858)	804 (751, 859)	825 (797, 883)
**East Asia**	28.5 (27.5, 29.6)	29.1 (28.0, 30.1)	28.9 (27.9, 30.0)	307 (303, 310)	361 (357, 365)	336 (332, 339)
**South Asia**	43.6 (42.2, 44.8)	45.8 (44.4, 47.1)	44.6 (43.3, 45.9)	576 (571, 581)	622 (617, 627)	599 (594, 603)
**Southeast Asia**	70.5 (68.8, 72.2)	73.3 (71.6, 75.0)	72.4 (70.7, 74.1)	783 (777, 788)	823 (818, 829)	807 (802, 813)
**West Asia**	62.8 (61.2, 64.3)	37.5 (36.3, 38.7)	51.1 (49.7, 52.5)	545 (540, 549)	563 (558, 568)	553 (549, 558)
**Total**	43.0 (41.8, 44.4)	42.6 (41.4, 43.9)	43.1 (41.8, 44.4)	505 (501, 510)	551 (547, 556)	530 (525, 534)
**Global**	37.4 (26.6, 51.3)	39.0 (27.8, 53.1)	38.3 (27.3, 52.3)	443 (398, 493)	501 (449, 561)	474 (426, 529)
**Total Blindness and Vision Loss**						
**Central Asia**	265 (262, 268)	284 (280, 286)	288 (285, 292)	8805 (8788, 8823)	8800 (8783, 8818)	9302 (9123, 9484)
**East Asia**	210 (207, 212)	243 (240, 246)	228 (225, 231)	8094 (8077, 8111)	9210 (9193, 9228)	8676 (8659, 8694)
**South Asia**	485 (481, 489)	543 (540, 553)	517 (512, 521)	14141 (14120, 14163)	15530 (15508, 15554)	14833 (14810, 14855)
**Southeast Asia**	379 (375, 383)	447 (442, 451)	418 (413, 422)	8132 (8115, 8149)	8988 (8970, 9006)	8596 (8579, 8614)
**West Asia**	311 (308, 315)	274 (271, 277)	337 (333, 340)	6822 (6807, 6838)	7389 (7823, 7856)	7103 (7087, 7119)
**Total**	349 (345, 352)	393 (389, 397)	376 (372, 379)	10351 (10332, 10370)	11463 (11443, 11483)	10927 (10908, 10947)
**Global**	264 (182, 372)	291 (201, 409)	278 (192, 392)	8246 (6907, 9727)	9096 (7606, 10679)	8687 (7270, 10218)

DALYs: Disability-adjusted life years.
